# Development and Evaluation of the Rapid and Sensitive RPA Assays for Specific Detection of *Salmonella* spp. in Food Samples

**DOI:** 10.3389/fcimb.2021.631921

**Published:** 2021-02-25

**Authors:** Liwei Zhao, Jianchang Wang, Xiao Xia Sun, Jinfeng Wang, Zhimin Chen, Xiangdong Xu, Mengyuan Dong, Ya-nan Guo, Yuanyuan Wang, Pingping Chen, Weijuan Gao, Yunyun Geng

**Affiliations:** ^1^Heibei Key Laboratory of Chinese Medicine Research on Cardiocerebrovascular Disease, Hebei University of Chinese Medicine, Shijiazhuang, China; ^2^Food Microbiology and Animal Quarantine Laboratory, Technology Center of Shijiazhuang Customs, Shijiazhuang, China; ^3^School of Public Health, Key Laboratory of Environment and Human Health, Hebei Medical University, Shijiazhuang, China

**Keywords:** *Salmonella*, *invA* gene, real-time RPA, lateral flow strip (LFS), isothermal amplification

## Abstract

*Salmonella* spp. is among the main foodborne pathogens which cause serious foodborne diseases. An isothermal real-time recombinase polymerase amplification (RPA) and lateral flow strip detection (LFS RPA) were used to detect *Salmonella* spp. targeting the conserved sequence of invasion protein A (*invA*). The Real-time RPA was performed in a portable florescence scanner at 39°C for 20 min. The LFS RPA was performed in an incubator block at 39°C for 15 min, under the same condition that the amplifications could be inspected by the naked eyes on the LFS within 5 min. The detection limit of *Salmonella* spp. DNA using real-time RPA was 1.1 × 10^1^ fg, which was the same with real-time PCR but 10 times higher than that of LFS RPA assay. Moreover, the practicality of discovering *Salmonella* spp. was validated with artificially contaminated lamb, chicken, and broccoli samples. The analyzing time dropped from 60 min to proximately 5–12 min on the basis of the real-time and LFS RPA assays compared with the real-time PCR assay. Real-time and LFS RPA assays’ results were equally reliable. There was no cross-reactivity with other pathogens in both assays. In addition, the assays had good stability. All of these helped to show that the developed RPA assays were simple, rapid, sensitive, credible, and could be a potential point-of-need (PON) test required mere resources.

## Introduction

*Salmonella* spp. is a Gram-negative bacterium belonging to the family of *Enterobacteriaceae*. *Salmonella* spp. is a major cause of the foodborne pathogen around the world (Nassib et al., 2003). It is widespread in nature and proliferates under ambient temperature with low nutritional demands (Li et al., 2013). *Salmonella* spp. infections attract much attention in public health especially in food safety. *Salmonella* spp. causes food poisoning, typhoid fever, gastrointestinal inflammation, and septicemia for both humans and animals (McGuinness et al., 2009; Rukambile et al., 2019). Currently, there are over 80 million cases of foodborne salmonellosis in the world (Li et al., 2019). Additionally, reports revealed that outbreaks caused by *Salmonella* spp. were largely associated with animal derived products such as poultry, egg, and chicken, and contamination is common in retail raw meats (Nassib et al., 2003; Foley and Lynne, 2008; Foley et al., 2008). An accurate and fast diagnosis is needed in order to prevent *Salmonella* spp. infections.

*Salmonella* spp. is currently detected in foods primarily through traditional laboratory methods. These traditional laboratory methods are inconvenient, time-consuming and takes over 3 days to obtain the result following multiple analytical steps (Li et al., 2019; Hong et al., 2020). Moreover, the complexity of the samples had a great effect on the bacterial morphology colony. The cross-reactivity among bacteria in *Enterobacteriaceae* restricted the specificity and sensitivity of the test (Nassib et al., 2003; Li et al., 2019). Upgrading molecular diagnostics provides powerful means for detecting *Salmonella* spp. in light of sensitivity and specificity. Currently, many nucleic acid amplification-based assays have gained popularity such as polymerase chain reaction (PCR), real-time PCR, multiplex PCR, reverse transcriptase PCR (RT-PCR), and loop-mediated isothermal amplification (LAMP) (Hirose et al., 2002; Yeh et al., 2002; Techathuvanan et al., 2010; Domesle et al., 2018). Real-time PCR is extensively applied for the quantitative detection of *Salmonella* spp. However, PCR requires sophisticated thermal cyclers with trained personnel which makes its use difficult in underequipped laboratories and low-resource field settings (Techathuvanan et al., 2011). PCR assays’ application is limited within the walls of the laboratories (Techathuvanan et al., 2011). In addition to the PCR assays, state-of-the-art isothermal amplification technologies such as LAMP, have been used for early and rapid detection of *Salmonella* spp. Simpler and more convenient techniques are required imperatively for the point-of-need (PON) diagnosis of *Salmonella* spp. in field conditions.

Recombinase polymerase amplification (RPA) acts as one of isothermal gene amplification techniques. RPA has the merit of amplification at a relatively low temperature (37–42°C) within 10–20 min (Piepenburg et al., 2006; Geng et al., 2018). The use of RPA-based methods has been proved to be a success in detecting pathogenic bacteria and viruses in clinical and food samples (Euler et al., 2012; Abd El Wahed et al., 2015; Wang et al., 2020). RPA-based methods have been designed to be a miniaturized diagnostic device that includes all the components for the RPA assay (Asiello and Baeumner, 2011). RPA assay was a rapid, stable, and promising assay for the on-site detection. The objective of the study was to develop the real-time and LFS RPA assays using the exo probe and nfo probe combined with lateral flow strip respectively as a way of rapidly detecting *Salmonella* spp. in food samples.

## Materials and Methods

### Bacterial Strains and DNA Extraction

A total of 34 common pathogenic bacteria strains were used to validate the techniques employed in this study (**Table 1**). These pathogenic bacteria were purchased from the American Type Culture Collection (ATCC), China Center of Industrial Culture Collection (CICC), China Center for Medical Culture Collection (CMCC) or isolated in the lab. All strains were reserved in the lab. Stock cultures were stored at −80°C in 0.8 ml of Nutrient broth (Beijing Land Bridge Technology Co., Ltd., Beijing, China) and 0.2 ml of 80% glycerol. The DNA templates were extracted using the TIANamp Bacteria DNA Kit (Tiangen, Beijing, China). These DNA samples were stored at −20°C before the assays.

**Table 1:** Bacterial strains and analytical specificity results for real-time RPA and LFS RPA assays.

Strain Name	Origin^1^	Real-time RPA^2^	LFS RPA^2^	Real-time PCR^2^
*Salmonella*	CICC 22956	+	+	+
*Salmonella aberdeen*	CMCC50786	+	+	+
*Salmonella dublin*	CMCC50761	+	+	+
*Salmonella taksony*	CMCC50359	+	+	+
*Salmonella typhimurium*	Isolated by lab	+	+	+
*Salmonella enteritidis*	Isolated by lab	+	+	+
*Salmonella paratyphi*	Isolated by lab	+	+	+
*Salmonella enterica*	Isolated by lab	+	+	+
*Enterobacter sakazakii*	ATCC 29544	−	−	−
*Staphylococcus aureus*	ATCC 6538	−	−	−
*Campylobacter jejuni*	ATCC 33291	−	−	−
*Vibrio parahaemolyticus*	ATCC 17802	−	−	−
*Pseudomonas aeruginosa*	ATCC 9027	−	−	−
*Vibrio vulnificus*	ATCC 27562	−	−	−
*Pseudomonas aeruginosa*	ATCC 9027	−	−	−
*Bacillus cereus*	CMCC 63301	−	−	−
*Listeria monocytogenes*	ATCC 19114	−	−	−
*Proteus mirabilis*	ATCC 35659	−	−	−
*Enterobacter aerogenes*	ATCC 13048	−	−	−
*Shigella sonnei*	ATCC 51592	−	−	−
*Bacillus licheniformis*	ATCC 14580	−	−	−
*Proteus pneumonia*	CMCC 49027	−	−	−
*Shigella boydii*	CMCC 51250	−	−	−
*Shigella flexneri*	CMCC51105	−	−	−
*Shigella flexneri*	CICC 21678	−	−	−
*Escherichia coli O157*:*H7*	CICC 21530	−	−	−
*Mannheimia haemolytica*	Isolated by lab	−	−	−
*Enterobacter cloacae*	Isolated by lab	−	−	−
*Citrobacter freundii*	Isolated by lab	−	−	−
*Streptococcus*	Isolated by lab	−	−	−
*Bacillus subtilis*	Isolated by lab	−	−	−
*Pseudomonas aeruginosa*	Isolated by lab	−	−	−
*Staphylococcus pasteuri*	Isolated by lab	−	−	−
*Escherichia coli*	Isolated by lab	−	−	−

^1^ATCC, American Type Culture Collection; CICC, China Center of Industrial Culture Collection; CMCC, China Center for Medical Culture Collection.

^2^+, positive results; −, negative results.

### RPA Primers and Probe

Nucleotide sequence data for *Salmonella* spp. strains from GenBank were aligned to identify conserved regions. Based on the reference sequences of different *Salmonella* spp. genotypes (accession numbers: AY594273, AY594271, DQ644632, DQ644633, EU348367, EU348368, JF951188, and JN982040), three pairs of primers targeting the conserved region of *invA* were designed (Rahn et al., 1992; Gonzalez-Escalona et al., 2009). RPA, Real-time RPA primers, and probes were then selected through testing the combination that yielded the highest sensitivity (**Table 2**). Primers and exo probes were synthesized by Sangon (Sangon, Shanghai, China).

**Table 2:** Primer and probe sequences for *Salmonella* spp. Real-time PCR, RPA, real-time RPA and LFS RPPA assays.

Method	Name^1^	Sequence 5´-3´Amplication	Size(bp)
Real-time RPA LFS-RPA	RPA-FP	GTCATTCCATTACCTACCTATCTGGTTGATTTCC	200
	RPA-RP	GCATCGGCTTCAATCAAGATAAGACGACTGGT	
	exo Probe	GTACTGGCGATATTGGTGTTTATGGGGTCGT-(FAM-dT)-THF-(BHQ1-dT)-ACATTGACAGAATCC-C3-spacer	
	nfo Probe	FAM-GTACTGGCGATATTGGTGTTTATGGGGTCGTT-THF-T-ACATTGACAGAATCC-C3-spacer	
Real-time PCR	PCR-FP	GAAGTTGAGGATGTTATTCGCAAAG	68
	PCR-RP	GGAGGCTTCCGGGTCAAG	
	Probe	JOE-CCGTCAGACCTCTGGCAGTACCTTCCTC-Eclipse	

^1^FP, Forward primer; RP, Reverse primer.

### Real-Time RPA Assay

Real-time RPA was accomplished in the tube with 50 μl reaction volume, including 40.9 μl of Buffer A (rehydration buffer), 2.0 μl of each RPA primers (***Sa***-exo-F and ***Sa***-exo-R, 10 μmol/L), 0.6 μl of exo probe (***Sa***-exo-P,10 μmol/L), and 2.5 μl of Buffer B (magnesium acetate, 280 mmol/L). Furthermore, 1 μl of genomic DNA was used for the specificity and sensitivity analysis, or 2 μl of sample DNA was used for the clinical sample diagnosis. In the process, the Genie III scanner device (OptiGene Limited, West Sussex, UK) and TwistAmpTM exo kit (TwistDX, Cambridge, UK) were applied.

### LFS RPA Assay

Moreover, the LFS RPA assay was performed according to the given instructions. The commercial TwistAmp™ nfo kit (TwistDX, Cambridge, UK) was used in the LFS RPA. The reactions were performed in a 50 μl volume with 29.5 μl of rehydration buffer with 2.5 μl of magnesium acetate (280 mM) included. Other components contained 420 nM RPA primer, 120 nM exo probe, and 1 μl of bacterial genomic DNA or 5 μl of sample DNA. The assay was performed in an incubator block at 39°C for 15 min and the lateral flow strips (Ustra Biotec GmbH, Germany) were employed to discover the RPA amplicons dual-labeled with FAM and biotin. Testing samples were considered positive when both the test line and the control line were visible. The testing sample was considered negative when the control line was visible. However, the sample was considered invalid when the control line was invisible.

### Real-Time PCR

Real-time PCR was performed on the ABI 7500 instrument in which premix Ex Taq TM (Takara Co., Ltd., Dalian, China) was employed (Geng et al., 2019). The reaction was performed as follows: 95°C for 30 s, followed by 35 cycles of 95°C for 10 s and then 60°C for 34 s. The sequences of the primers and probes used for real-time PCR were listed in **Table 2**. The reporter and fluorescence quencher were marked with 6-FAM (6-CarboxyFluorescein) and BHQ1 (Black Hole Quencher 1) respectively.

### Analytical Specificity and Analytical Sensitivity Analysis

For the food security, the real-time RPA and LFS RPA assays were evaluated to amplify the nucleic acid of some important pathogens. Five independent reactions were performed.

The genomic DNA of *Salmonella* spp. varying from 1.1 × 10^8^ to 1.1 × 10^0^ fg was diluted in nuclease-free water for the analytical sensitivity analysis of the RPA. One microliter of each DNA dilution was amplified by both RPA assays to determine the limit of detection (LOD). The culture of *Salmonell*a spp. was diluted in sterile water (ranging from 1.4 × 10^7^ to 1.4 × 10^0^ CFU) and counted by plate counting in 37°C overnight. The sensitivity of the real-time RPA and LFS RPA method were assessed with *Salmonella* spp. in pure culture. The analytical sensitivity analysis was repeated for five times.

### Validation With Artificially Contaminated Samples

The pure colony of *Salmonella* spp. was picked into a tube containing 1 ml sterile saline. The solution was vortexed for 30 s and the turbidity was measured to 1.00 using a turbidimeter. Sterile saline was used for 10-fold gradient dilution until it attained 10^−8^ dilution. With 10^−5^, 10^−6^, and 10^−7^ diluents of 200 μl on the chromogenic medium of *Salmonella* spp., the initial concentration of the pure culture bacteria was calculated using three parallels.

Commercially available chicken/lamb/broccoli were purchased from a local supermarket free of *Salmonella* spp. to assess the potential use and suitability of the developed RPA assays. Testing of the samples was done according to the Chinese national standard (GB 4789.4-2016). A total of 4, 14, and 59 CFU/25g of *Salmonella* spp. with chicken, lamb, and broccoli respectively, were added into a sterile stomaching bag containing 225 ml Nutrient broth. These samples were mixed well to get homogenous samples and incubated for 6 or 8 h at 37°C to increase the bacterial concentrations to attain detectable levels. The Bacterial genomic DNA extraction, the real-time RPA, and LFS RPA reactions were performed, and each experiment was repeated for no less than three times to attain results.

## Results

### Analytical Specificity and Sensitivity of the Real-Time RPA and LFS-RPA Assay

**Figure 1:**
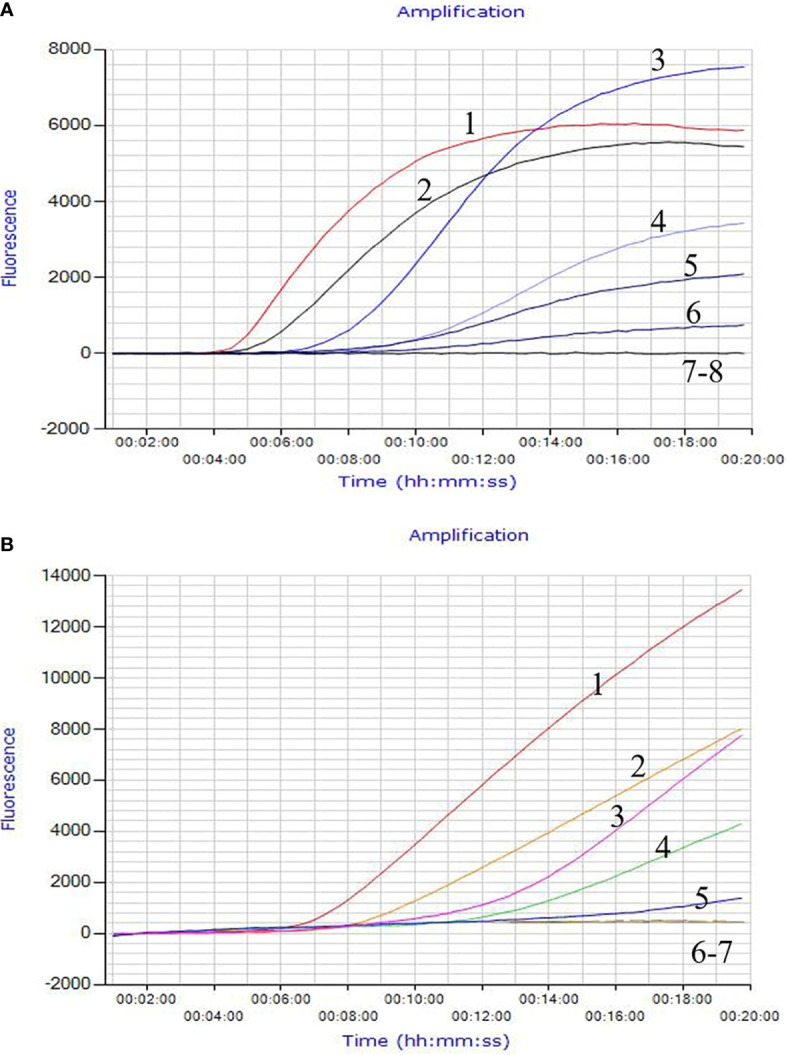
Analytical Sensitivity of the real-time RPA assay. The LOD of the real-time RPA was 1.1 × 10^1^ fg/μl of *Salmonella* spp. standard DNA and 1.4 × 10^2^ CFU/ml for bacteria in pure culture. Fluorescence development over time using a dilution range of 1.1 × 10^6^–1.1 × 10^0^ fg of *Salmonella* spp. genomic DNA. For **(A)**: Curve 1, 1.1 × 10^6^ fg; Curve 2, 1.1 × 10^5^ fg; Curve 3, 1.1 × 10^4^ fg; Curve 4, 1.0 × 10^3^ fg; Curve 5, 1.1 × 10^2^ fg; Curve 6, 1.1 × 10^1^ fg; Curve 7, 1.1 × 10^0^ fg; Curve 8, ddH_2_O. For **(B)**: Curve 1, 1.4 × 10^6^ CFU; Curve 2, 1.4 × 10^5^ CFU; Curve 3, 1.4 × 10^4^ CFU; Curve 4, 1.4 × 10^3^CFU; Curve 5, 1.4 × 10^2^ CFU; Curve 6, 1.4 × 10^1^CFU; Curve 7, 1.4 × 10^0^ CFU.

**Figure 2:**
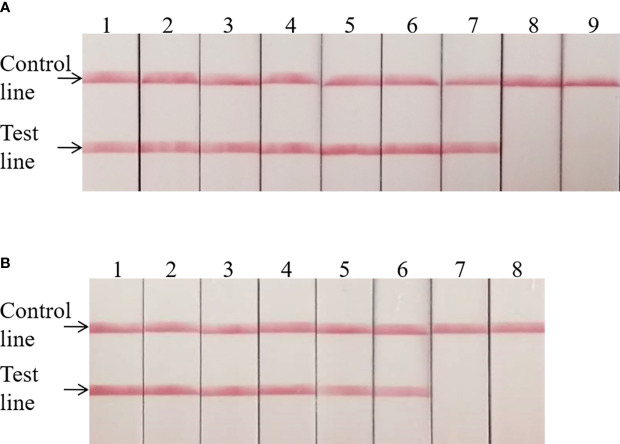
Analytical sensitivity of the LFS RPA assay. The LOD of the LFS RPA method was 1.1 × 10^2^ fg for genomic DNA and 1.4 × 10^2^ CFU/ml for bacteria in pure culture. For **(A)**: Sample 1, 1.1 × 10^8^ fg; Sample 2, 1.1 × 10^7^fg; Sample 3, 1.1 × 10^6^ fg; Sample 4, 1.1 × 10^5^ fg; Sample 5, 1.1 × 10^4^ fg; Sample 6, 1.1 × 10^3^ fg; Sample 7, 1.1 × 10^2^ fg; Sample 8, 1.1 × 10^1^ fg; Sample 9, 1.1 × 10^0^ fg. For **(B)**: Sample 1, 1.4 × 10^7^ CFU; Sample 2, 1.4 × 10^6^ CFU; Sample 3, 1.4 × 10^5^ CFU; Sample 4, 1.4 × 10^4^ CFU; Sample 5, 1.4 × 10^3^ CFU; Sample 6, 1.4 × 10^2^ CFU; Sample 7, 1.4 × 10^1^ CFU; Sample 8, 1.4 × 10^0^ CFU.

The *invA* gene coding of the invasion protein of *Salmonella* spp. is the most used specific gene for the discovery of many different *Salmonella* spp. serotypes. The RPA primers and probes were designed according to the *invA* gene of *Salmonella* spp. in this study. Both RPA assays provide excellent results at 39°C within 20 min. This was faster than any other common nucleic acid amplification method. The analytical sensitivity of RPA methods was evaluated by employing *Salmonella* spp. genomic DNA and bacterial pure culture as templates from 1.1 × 10^8^ to 1.1 × 10^0^ fg and from 1.4 × 10^7^ to 1.4 × 10^0^ CFU. The data on the analytical sensitivity of RPA methods was presented in **Figures 1A**, **2A**. The detection limit (LOD) of real-time RPA was 1.1 × 10^1^ fg similar to that of real-time PCR (the data was not shown) (**Figure 1A**). The limit of detection of the LFS RPA method was 1.1 × 10^2^ fg for genomic DNA (**Figure 2A**), 10-times lower compared with the real-time PCR. The LOD of the real-time RPA and LFS RPA was 1.4 × 10^2^ CFU for bacteria in pure culture (shown in **Figures 1B**, **2B**).

Regarding specificity, only amplification signal was observed at the control line with *Salmonella* spp. and no cross-detection of other pathogens were shown in both real-time RPA and LFS-RPA assays (**Table 1**). Five independent reactions were repeated and similar results were obtained. This manifests the high specificity of the assays.

### Evaluation With Artificially Contaminated Samples After Enrichment

The diagnostic performance of the developed RPA assays was compared with other detection approaches and was shown in **Table 3**. This was done while detecting *Salmonella* spp. in artificially contaminated food samples. The National Standard GB 4789.4-2016 method was used as a reference to guarantee that the food samples were successfully contaminated. However, after 6-h enrichment of food samples contaminated with 4 CFU/25 g of *Salmonella* spp., no *Salmonella* spp. was discovered through any of the detection methods. This signaled no false-positive results from samples containing low levels of *Salmonell*a spp. All contaminated food samples were detected and showed increasing values of CFU/25 g of spiked samples and enrichment time. However, lamb contaminated with 14 CFU/25 g of *Salmonella* spp. was different from the other contaminated food samples and was enriched for 6 h. Furthermore, a diagnostic agreement of 100% with real-time PCR and the traditional method was indicated in the developed real-time RPA and LFS RPA assays. Moreover, the speed of the RPA assays outstripped. The time required to attain positive results with real-time RPA and LFS RPA was 5–12 min and 15 min respectively. Real-time PCR with CT values at 19.34–34.01 required approximately 20–35 min. Therefore, the results demonstrated that, with the equal sensitivity, the real-time and LFS RPA assays was faster than the real-time PCR.

**Table 3:** The comparison of reaction time of different methods in contaminated foods.

Food samples	Spiked samples^1^ (CFU/25g)	Enrichment time(h)	Real-time RPA (min:ss)	LFS-RPA^2^ (min)	Real-timePCR^3^ (Ct)	GB4789.4-2016	Viable cell counts (CFU/g)
Lamb	4	6	−	−	−	−	0
		8	12:18	15(+)	33.24	+	4.7 × 10^3^
	14	6	−	−	−	−	0
		8	11:44	15(+)	33.38	+	5.1 × 10^3^
	59	6	12:04	15(+)	33.78	+	4.6 × 10^3^
		8	10:15	15(+)	30.58	+	7.8 × 10^3^
Broccoli	4	6	−	−	−	−	0
		8	10:09	15(+)	30.02	+	6.2 × 10^3^
	14	6	6:55	15(+)	28.47	+	1.3 × 10^3^
		8	5:16	15(+)	19.34	+	5.3 × 10^5^
	59	6	6:33	15(+)	25.46	+	1.8 × 10^4^
		8	5:39	15(+)	20.58	+	1.8 × 10^5^
Chicken	4	6	−	−	−	−	0
		8	11:54	15(+)	33.49	+	3.8 × 10^4^
	14	6	12:06	15(+)	34.01	+	6.3 × 10^3^
		8	6:14	15(+)	25.18	+	2.8 ×10^5^
	59	6	11:22	15(+)	33.28	+	3.9 × 10^4^
		8	5:54	15(+)	22.39	+	3.7 × 10^5^

^1^CFU, colony-forming units; ^2^Ct, Cycle threshold; ^3^+, detected; −, not detected.

Total bacterial count, 1.5 × 10^7^ CFU/g and coliform group, 3.1 × 10^4^ CFU/g in Lamb;

Total bacterial count, <1.0 × 10 CFU/g and coliform group, <1.0 × 10 CFU/g in Broccoli;

Total bacterial count, 3.7 × 10^2^ CFU/g and coliform group, <1.0 × 10 CFU/g in Chicken.

## Discussion

The disease induced by foodborne pathogens remains a major public health issue worldwide despite ongoing measurements to ensuring food safety. *Salmonella* spp. frequently leads to infections and worldwide outbreaks accounting for huge economic costs and life loss every year (Foley and Lynne, 2008). Rapid and reliable diagnostic techniques play an important part to efficiently detect *Salmonella* spp. from contaminated specimens and make appropriate measures for preventing and controlling the risk of *Salmonella* spp. infection as early as possible.

The real-time RPA and LFS RPA assays are good choices for detecting *Salmonella* spp. as demonstrated in this report. These assays are specific, sensitive, and simple to perform. In the specificity analysis, both the real-time RPA and LFS RPA only amplified the genomic DNA of *Salmonella* spp. used in the study. This indicated high specificity of these assays. However, other more *Salmonella* strains are needed to further examine the cross-reactivity of these RPA assays. The real-time RPA had equal sensitivity (limit of detection) as real-time PCR in this study. This was 10 times higher than the LFS assay. However, it is possible for a varied reaction mechanism and enzyme kinetics between the different methods. The reaction time of RPA assays was much shorter than real-time PCR. The diagnostic performances of the developed real-time RPA and LFS RPA assays has been further assessed. These assays were proved to be a success in the detection of the artificially contaminated food samples, and performed better than the real-time PCR in light of the detecting speed. However, a pre-enrichment step was necessary when the level of pathogen contamination was low. A similar ideal result was obtained using the direct water boiling method to extract the bacterial DNA as the template of RPA reaction. Direct boiling method was used to extract the *Salmonella* spp. genomic DNA as the template of the RPA reaction. The LFS strip were combined to facilitate the detection of *Salmonella* spp. at quarantine stations, ports, or the site of outbreak by the PRA assay based on nfo-probe.

RPA was first introduced in 2006 and represented an innovative DNA isothermal detecting technology beyond the reach of PCR or traditional culture-based methods (Piepenburg et al., 2006; James and Macdonald, 2015). RPAs have successfully been practiced in the discovery of pathogenic bacteria (Hong et al., 2020), fungus (Ahmed et al., 2014), and viruses (Boyle et al., 2013). The reagents in RPA are available in lyophilized form for long-term storage and are conveniently transported even without cold-chain (Wang et al., 2020). Moreover, under the prerequisite that the testing results were visible, the real-time RPA assay was accomplished on the user-friendly PON (point of need) detection platform (Genie III) with battery power. The developed LFS RPA assay only needed a simple incubator block. Therefore, these two pieces of equipment were portable, lightweight, and less expensive than the equipment for LAMP/PCR. As other isothermal DNA amplification methods, Loop-mediated isothermal amplification (LAMP) and the cross-priming amplification assay (CPA) have been adopted for rapidly and sensitively detecting *Salmonella* spp. Both RPA assays have the merits of amplification at a relatively lower temperature and within shorter time than that of LAMP and CPA assays. Both RPA reactions could be done at 37–42°C within 10–20 min. However, the optimum time and temperature were approximately 60 min and above 60°C respectively which were required for LAMP and CPA (Domesle et al., 2018; Wang et al., 2018). Moreover, several reports have shown that RPA is tolerant to mismatches, background DNA, and most of PCR inhibitors (Daher et al., 2016; Li et al., 2019; Liu et al., 2019). All of these outstanding characteristics make the assays readily suitable for the field, PON (point-of-need), or diagnosis of infectious diseases with poor environmental resources.

In conclusion, the current study proved that, the developed RPA assays with high specificity and sensitivity was convenient, rapid, and reliable for *Salmonella* spp. detection. In addition, the simple devices and easy operation protocol helped to improve the efficiency of detection. Among the isothermal amplification techniques, real-time RPA and LFS RPA assays play an outstanding role in preventing and controlling of *Salmonella* spp. especially in the settings with limited resources.

## Data Availability Statement

The datasets presented in this study can be found in online repositories. The names of the repository/repositories and accession number(s) can be found in the article/supplementary material.

## Author Contributions

YG, JCW, and WG designed and conducted the experiment. LZ, XS, JFW, XX, MD, Y-nG, YW, and PC performed the experiments and analyzed the data. YG drafted the manuscript. All authors contributed to the article and approved the submitted version.

## Funding

This work was supported by the program of Traditional Chinese Medicine Scientific Research foundation in Hebei Administration of Traditional Chinese Medicine (2020142, Hebei, China), the Project of Excellent Young Teacher Fundamental Research (YQ2019003) and Doctoral Foundation (BSZ2019009) of Hebei University of Chinese Medicine.

## Conflict of Interest

The authors declare that the research was conducted in the absence of any commercial or financial relationships that could be construed as a potential conflict of interest.
